# Targeted Mutagenesis of *Arabidopsis thaliana* Using Engineered TAL Effector Nucleases

**DOI:** 10.1534/g3.113.007104

**Published:** 2013-10-01

**Authors:** Michelle Christian, Yiping Qi, Yong Zhang, Daniel F. Voytas

**Affiliations:** * Department of Genetics, Cell Biology, and Development and Center for Genome Engineering, University of Minnesota, Minneapolis, Minnesota 55455; † Department of Biotechnology, School of Life Sciences and Technology, University of Electronic Science and Technology of China, Chendu 610054, China

**Keywords:** *Arabidopsis thaliana*, nonhomologous end-joining, TAL effector nuclease, targeted mutagenesis

## Abstract

Custom TAL effector nucleases (TALENs) are increasingly used as reagents to manipulate genomes *in vivo*. Here, we used TALENs to modify the genome of the model plant, *Arabidopsis thaliana*. We engineered seven TALENs targeting five Arabidopsis genes, namely *ADH1*, *TT4*, *MAPKKK1*, *DSK2B*, and *NATA2*. In pooled seedlings expressing the TALENs, we observed somatic mutagenesis frequencies ranging from 2–15% at the intended targets for all seven TALENs. Somatic mutagenesis frequencies as high as 41–73% were observed in individual transgenic plant lines expressing the TALENs. Additionally, a TALEN pair targeting a tandemly duplicated gene induced a 4.4-kb deletion in somatic cells. For the most active TALEN pairs, namely those targeting *ADH1* and *NATA2*, we found that TALEN-induced mutations were transmitted to the next generation at frequencies of 1.5–12%. Our work demonstrates that TALENs are useful reagents for achieving targeted mutagenesis in this important plant model.

In *Arabidopsis thaliana*, reverse genetic approaches, such as T-DNA and transposon insertional mutagenesis and targeting-induced local lesions in genomes (TILLING), have enabled the recovery of mutations in a variety of genes (Feldmann 1991; Jones and Dean 1993; McCallum *et al.* 2000; Alonso *et al.* 2003; Henikoff *et al.* 2004). These mutagenesis approaches, however, are labor-intensive and provide little control over where the mutations occur. Moreover, many genes are difficult to target (*e.g.*, small microRNA genes and duplicated gene arrays), and there are still thousands of genes for which no mutations have yet been recovered (Alonso *et al.* 2003). Whereas other reverse genetics approaches, such as RNAi and artificial microRNAs, provide specificity for intended targets, it is sometimes difficult to achieve a null phenotype by knocking down target gene expression (Chuang and Meyerowitz 2000; Schwab *et al.* 2006). The ability to make specific, targeted gene knockouts would clearly be a valuable addition to the repertoire of genetic tools available for Arabidopsis.

Recent efforts to achieve targeted mutagenesis in plants have focused on the use of sequence-specific nucleases, which include zinc finger nucleases (ZFNs), meganucleases, and transcription activator-like effector nucleases (TALENs) (Voytas 2012). CRISPR/Cas systems also likely hold promise for plant genome engineering (Cong *et al.* 2013; Mali *et al.* 2013). All of these nucleases work by introducing a targeted DNA double-strand break in the plant genome that is repaired by one of two pathways. For repair by nonhomologous end-joining (NHEJ), the break is simply rejoined, sometimes imprecisely, and this can result in insertions or deletions at the break site that disrupt gene function (Waterworth *et al.* 2011). Repair by homologous recombination relies on sequence homology provided by a donor DNA molecule, and information from the donor is copied to the broken chromosome. Homologous recombination allows a wide variety of DNA sequence alterations to be made at or near the break site.

Previous work using ZFNs enabled the recovery of plants with mutations in endogenous Arabidopsis genes (Osakabe *et al.* 2010; Zhang *et al.* 2010). One challenge in using ZFNs, however, is that they are difficult to engineer to recognize new target sites (Maeder *et al.* 2008; Ramirez *et al.* 2008). The DNA binding domain of TALENs, however, is easy to engineer to have new DNA sequence specificities (Cermak *et al.* 2011; Reyon *et al.* 2012). TALENs have been used to modify genes in tobacco and Arabidopsis protoplasts (Mahfouz *et al.* 2010; Cermak *et al.* 2011; Zhang *et al.* 2011). More recently, TALENs delivered by Agrobacterium have successfully created mutations in rice, barley, and Brachypodium (Li *et al.* 2012; Shan *et al.* 2013; Wendt *et al.* 2013). In all three cases, somatic mutagenesis was observed in plant tissue expressing the TALENs. One group successfully recovered modified rice plants that were resistant to a plant pathogen because of a TALEN-induced mutation (Li *et al.* 2012).

In this study, we used TALENs to create targeted, heritable mutations in Arabidopsis genes. Our goal was to implement TALEN-based mutagenesis using the most commonly practiced transformation technique, namely the robust Agrobacterium-mediated floral dip transformation method. We stably integrated TALEN expression constructs and induced expression during germination with an estrogen-inducible promoter. Using this so-called *in planta* mutagenesis strategy, we were able to recover TALEN-induced mutations in endogenous genes in 1.5–12% of the progeny.

## Materials and Methods

### TALEN design, validation, and expression in plants

TALEN target sites were identified using the TAL effector-nucleotide targeter (TALE-NT) web site (Doyle *et al.* 2012). All target sites retained a T at the −1 position. Corresponding TAL effector arrays were constructed by Golden Gate cloning as previously described (Cermak *et al.* 2011). Information for all of the TAL effector arrays and target sites is listed in Supporting Information, Table S1. TALENs were assembled in vectors with the truncated N152/C63 backbone architecture (pZHY500 and pZHY501) (Zhang *et al.* 2013). Fully assembled TAL effector arrays and surrounding N-terminal and C-terminal regions were cloned into the gateway-compatible entry clone, pZHY013, using *Xba*I and *Bam*HI (generating pMC89–95, pZHY013-TALN1–4). pZHY013 contains the EL/KK *Fok*I heterodimers and a viral T2A ribosomal skipping motif that allows translation of both the left and right TALENs from a single transcript (Halpin *et al.* 1999). An estrogen-inducible, gateway-compatible expression vector, pFZ19, was used to generate transgenic Arabidopsis plants (Zhang *et al.* 2010). A gateway LR cloning reaction was performed by recombining the entry clones with pFZ19 to generate the vectors pMC105 (*ADH1*), 107 (*TT4*), 108 (*MAPKKK1*), pTALEN5+6 (*DSK2Ba*), pTALEN7+8 (*DSK2Bb*), pTALEN13+14 (*NATA2a*), pTALEN15+16 (*NATA2b*), and pTALEN-*GLL22* (*GLL22*a and *GLL22*b; accession ID *At1g54000* and *At1g54010*). In the final T-DNA expression vectors, the XVE promoter drives expression of both the left and right TALENs (Zuo *et al.* 2000). Several TALEN pairs were also cloned into T-DNA expression vectors containing a constitutive 35S promoter. Entry clones pMC89 and pZHY013-TALN1–4 were recombined with pMDC32, a 35S T-DNA expression vector, using LR clonase to generate pMC100–102 (TALENs *ADH1*, *TT4*, *MAPKKK1*) and p35STALEN1–4 (TALENs *DSK2Ba/b* and *NATA2a/b*).

### Testing TALEN activity in plants

Stably transformed Arabidopsis lines were generated by the floral dip method (Clough and Bent 1998). T-DNA expression vectors were transformed into *Agrobacterium tumefaciens* strain GV3101. Floral dip transformation was conducted using the Columbia ecotype. Seeds from dipped plants were collected and plated onto solid Murashige and Skoog (MS) medium containing 25 mg/L hygromycin to select for transformants with the transgene and 10–20 μM 17β-estradiol to induce TALEN expression (for those plants with XVE TALEN constructs). The MS plates containing seeds were placed at 4° for 3–4 d in the dark for stratification. Plates were then moved to a growth chamber and grown under a regime of 16 hr light/8 hr dark at 21° in a growth chamber.

### T7 endonuclease and PCR digestion assays to detect somatic mutations

To assess frequencies of somatic mutagenesis, genomic DNA was extracted from 5–10 pooled T1 seedlings for each TALEN tested. The T7 endonuclease assay was then performed using a modification of a previously described protocol (Kim *et al.* 2009). For each reaction, 400 ng purified PCR product was denatured and reannealed in NEBuffer 2 (New England Biolabs) in a thermocycler using the following regime: 95° for 5 min; 95–85° at −2°/s; 85–25° at −0.1°/s; and hold at 4°. The reaction was treated with 10 U of T7 endonuclease I at 37° for 30 min in a final volume of 20 μl. Reactions were then column-purified, visualized on a 2% sodium borate gel, and quantified by densitometry as described.

For the *NATA2b* TALEN, a 25-μl PCR reaction was performed to amplify target sites using 50–200 ng genomic DNA from pooled seedlings; 10–15 μl of the PCR reaction was subsequently digested with the restriction enzyme *Nsp*I, which cuts in the spacer of the TALEN target site. PCR products resistant to *Nsp*I digestion result from loss of the *Nsp*I site because of TALEN cleavage and imprecise repair by NHEJ. Digestions were then run on 2% agarose gels and viewed under UV light. Bands were quantified by densitometry using ImageJ software (Schneider *et al.* 2012) and analyzed as previously described (Guschin *et al.* 2010). The fraction of undigested PCR product relative to digested product provided an estimate of the somatic mutation frequency.

Additional analysis was performed on PCR products from the *ADH1* and *TT4* loci. Regions surrounding the *ADH1* and *TT4* target sites were amplified by PCR using the same primers as in the T7 endonuclease assay. The PCR products were cloned into TOPO (Invitrogen) vectors, and 84 *ADH1* and 47 *TT4* clones were randomly selected for DNA sequencing. The number of sequences with mutations in the TALEN spacer sequence was divided by the total number of sequencing reads for each locus to yield the somatic mutation frequency. For sequence analysis of the remaining TALEN targets, namely *MAPKKK1*, *DSK2Ba/b*, *NATA2a/b*, and *GLL22*, pooled genomic DNA was first predigested with the appropriate restriction enzyme, followed by PCR amplification and a second digestion reaction. Resistant bands from these enrichment PCR assays were purified and cloned into TOPO vectors. Several clones were randomly chosen for sequence analysis.

### Screening for germline transmission of mutations

To obtain germinal events, rosette leaves were collected from individual T1 parental lines at the 6–10-leaf stage and genomic DNA was isolated. Limited-cycle PCR followed by restriction digestion was performed, and digested bands were visualized on 2% agarose gels. The amount of digestion-resistant product was quantified by densitometry using ImageJ software and expressed as a percentage of the total amplicon (Schneider *et al.* 2012). As described in Results, progeny from plants showing high frequencies of somatic mutagenesis were then screened for heritable mutations.

To screen for *ADH1* mutant progeny, seeds were collected from 11 transgenic plants carrying the *ADH1* TALEN XVE constructs for which transgene expression had been induced. Genomic DNA from >133 sibling plants (originating from four individual T1 lines) was subjected to limited-cycle, high-fidelity PCR (Zhang *et al.* 2010). PCR products were digested with *Pfl*FI. Mutations in PCR reactions for which a resistant band was detected were confirmed by DNA sequencing. The same protocol was followed to recover inherited mutations at a second locus, *NATA2b*. Seeds from 18 T1 plants carrying the *NATA2b* 35S TALEN construct were collected. Genomic DNA from 189 sibling plants originating from two distinct parental lines were assayed by PCR and digested with *Nsp*I. Four mutant plants were recovered and deletions within the TALEN target site were confirmed by sequencing the PCR product.

## Results

### Design of TALENs targeting endogenous Arabidopsis genes

**Figure 1:**
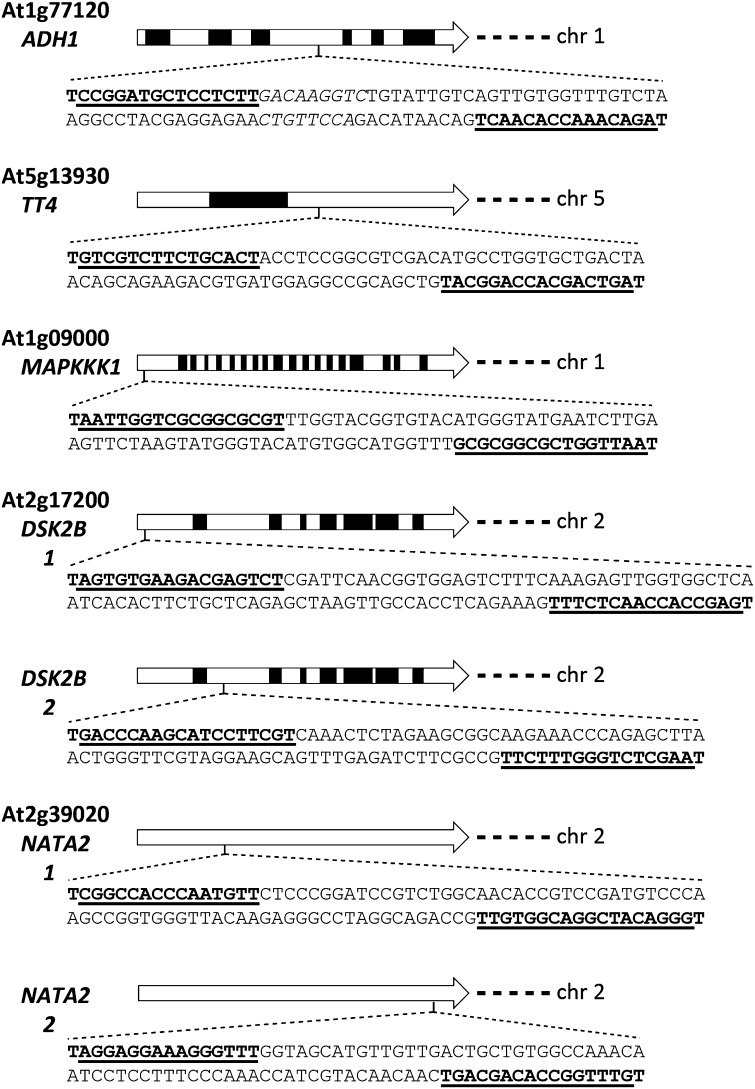
TAL effector nuclease (TALEN) sites in the coding sequences of target Arabidopsis genes. Arrows depict gene models; introns are in black. Locations of TALEN target sites are indicated, and their DNA sequences are provided below the gene model. Sequences recognized by TALENs are underlined and in bold. The locus name, gene name, and chromosomal location of the target genes are also provided.

We designed seven different TALEN pairs targeting unique sequences in five Arabidopsis genes (Figure 1). Target sites were identified using the publicly available TALE-NT program. Each target had a T nucleotide at the −1 position (Doyle *et al.* 2012). The number of repeats in each TAL effector array ranged from 15 to 18, and the length of the spacers within the target sites were between 14 and 22 bp (Table S1). One TALEN pair was designed to recognize the same target sequence in two homologous genes organized in tandem.

We initially engineered four TALENs using the so-called *Bam*HI backbone architecture, which has long N-terminal and C-terminal regions derived from the native TAL effector that flank the DNA binding domain (pTAL-*Bam*HI) (Christian *et al.* 2010; Cermak *et al.* 2011). Although TALENs made with the *Bam*HI backbone showed consistently high activity in a yeast-based activity assay (Figure S1A), we found that this activity was not always reflective of mutagenesis efficiencies in somatic tissue of whole plants (Figure S1B). Among three TALENs made with the *Bam*HI backbone, only the *ADH1* TALEN showed detectable mutagenesis activity *in planta* (Figure S1B) that was confirmed by DNA sequencing (Figure S1C). A second TALEN backbone architecture with enhanced nuclease activity was reported that lacks the first 152 aa at the N-terminus of the TALE effector and only has 63 aa after the last repeat in the array (designated N152/C63) (Miller *et al.* 2011). We redesigned our TALEN pairs using this truncated architecture and used them for all subsequent experiments.

### TALENs induce mutations at endogenous loci in somatic plant tissue

**Figure 2:**
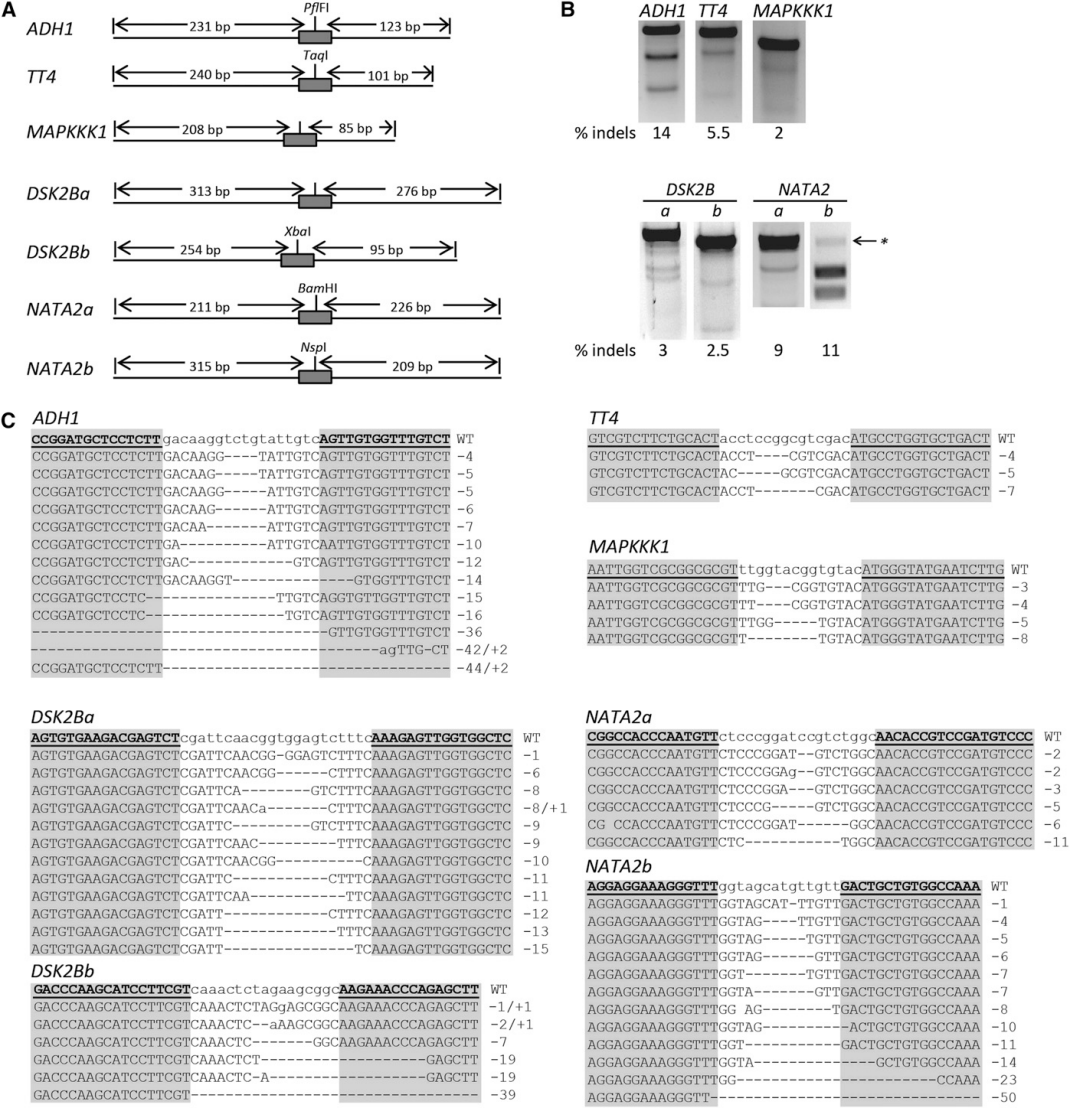
Detection of nonhomologous end-joining (NHEJ)-induced mutations in somatic cells of plants expressing TAL effector nucleases (TALENs). (A) Schematic of the PCR amplicons and TALEN target sites (gray rectangles). Lengths of expected cleavage products after digestion with T7 endonuclease are indicated. Restriction enzyme sites within the TALEN spacer sequences are also shown. Loss of these restriction sites was used to measure TALEN activity (*NATA2* in panel B, lane b and Figure 3). (B) T7 endonuclease assays reveal TALEN-induced mutations in pooled leaf samples. Genomic DNA was isolated from 5 to 10 pooled seedlings collected 7–10 days after induction of TALEN expression. Mutagenesis frequencies (% indels) were calculated by measuring band intensities. Treatment with T7 endonuclease I yielded unclear results for sample *NATA2b*, so PCR amplification followed by digestion with *Nsp*I was used to detect target site mutations. A black arrow with an asterisk indicates the position of the DNA band that is resistant to digestion because of TALEN-induced mutations in the enzyme recognition sequence. (C) DNA sequencing reveals TALEN-induced mutations. Left and right TALEN target sequences are underlined and in bold in the wild-type sequence; gray shading denotes the TALEN target sequences in the clones. Deletions in the sequence alignments are represented by dashes; inserted bases are in lowercase. The lengths of insertions (+) or deletions (−) are indicated to the right of the sequences.

We tested the ability of TALENs to induce mutations at native sites in five genes in Arabidopsis. TALEN pairs were introduced into plants (ecotype Columbia) using the Agrobacterium-mediated floral dip transformation method (Clough and Bent 1998). TALEN expression was controlled by an estrogen-inducible promoter, which we previously used in our laboratory for ZFN mutagenesis (Zhang *et al.* 2010). Briefly, seeds were collected from plants transformed with Agrobacterium carrying the TALEN expression construct (T0 plants) and plated on MS media with hygromycin and β-estradiol to select for the transgene and to induce TALEN expression, respectively. Primary transgenic seedlings (T1) grown on MS media were pooled and genomic DNA was assessed using two previously described assays to detect NHEJ-induced mutations (Lloyd *et al.* 2005; Kim *et al.* 2009). One assay required the presence of a restriction enzyme site in the spacer of the TALEN target (Lloyd *et al.* 2005; Zhang *et al.* 2010). Following TALEN cleavage, restriction sites are often destroyed by NHEJ-induced mutations. Mutated sequences were identified by PCR-amplifying the target site and then quantifying the fraction of amplicons resistant to enzyme digestion. The percentage of digested amplicons provides an estimate of the somatic mutagenesis frequency. If an appropriate restriction site was not available, then the T7 endonuclease I (T7EI) assay was used (Kim *et al.* 2009). T7EI recognizes and cleaves mismatched heteroduplex DNA. Genomic DNA was isolated from pooled seedlings, and regions surrounding the targets for seven TALENs were amplified by PCR. The amplicons were denaturated and annealed to form homoduplexes and heteroduplexes. T7EI treatment cleaves the heteroduplexes, and the digested fragments were quantified by densitometry. The fraction of digested amplicons provided an estimate of somatic mutation frequency. All TALEN pairs were found to be active at their endogenous targets (Figure 2). The somatic mutation frequency of pooled samples ranged from 2% (for the *MAPKKK1* TALENs) to 14% (for the *ADH1* TALENs) (Figure 2B). No activity was detected in samples in which TALEN activity was not induced or absent.

To confirm the presence of NHEJ-induced mutations at the TALEN target sites, PCR amplicons were cloned and sequenced. Mutations within the spacer sequence of the targets confirmed that TALEN-induced breaks resulted in imprecise repair (Figure 2C). Efficiencies of the *ADH1* and *TT4* TALENs were 15% (13 of 87 sequenced clones) and 7% (3 of 43 sequenced clones), respectively. This corroborated the frequencies of somatic mutagenesis observed for these TALENs using the T7EI assay. The recovered mutations contained small deletions ranging from 1 to 50 bp in length. The TALEN pairs targeting exons one and two in the *DSK2B* gene showed the lowest levels of activity, namely approximately 3%. Taken together, these results indicate that TALENs can create targeted mutations in a number of endogenous genes in Arabidopsis.

### TALEN-induced mutations are transmitted to the next generation

NHEJ-induced mutations that occur in cells of the L2/L3 layers of the shoot apical meristem should be incorporated into the germline (Furner and Pumfrey 1992). To determine whether TALEN-induced mutations can be transmitted germinally, we first identified parental transgenic lines with high frequencies of somatic mutagenesis. We collected leaf tissue from individual transgenic T1 plants expressing TALENs and performed the aforementioned PCR and restriction enzyme digestion assay that identifies mutations through loss of a restriction enzyme recognition sequence within the TALEN target site.

**Figure 3:**
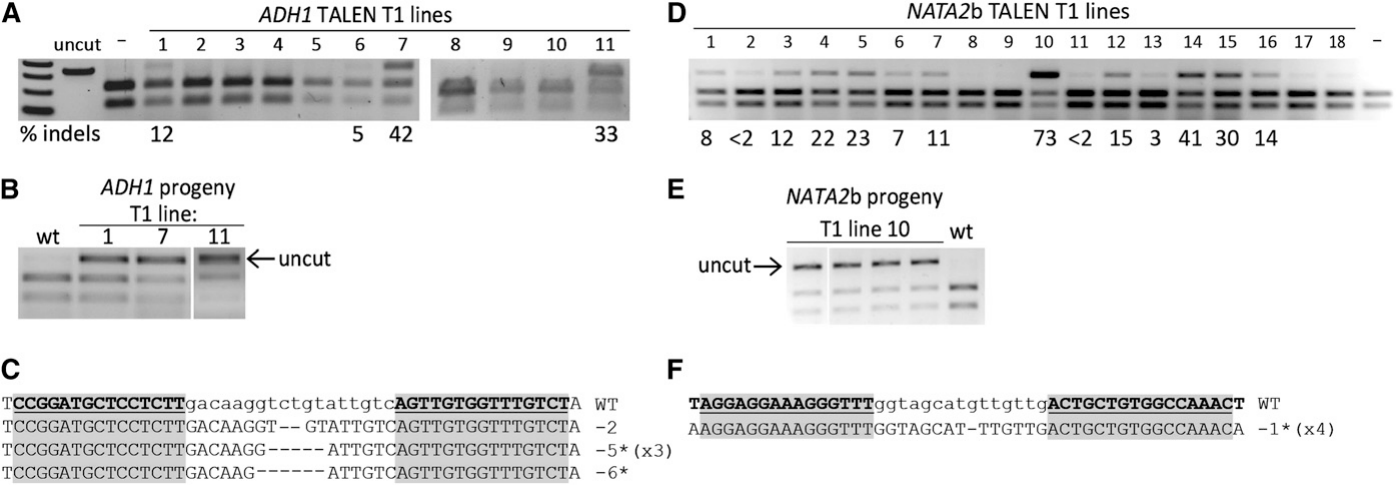
Germinal transmission of TAL effector nuclease (TALEN)-induced mutations. (A, D) Somatic mutagenesis frequencies detected by the PCR and restriction enzyme digestion assay in individual T1 plant lines expressing the *ADH1* (A) and *NATA2b* TALENs (D). Genomic DNA from 11 *ADH1* T1 plants and from 18 *NATA2b* plants was analyzed by PCR amplifying the target locus and then digesting with *Pfl*FI (*ADH1*) or *Nsp*I (*NATA2b*). Resistant bands in samples 1, 6, 7, and 11 are equal in size to the uncut product, indicating mutations present in the TALEN target sites. The intensity of the resistant band was measured and used to calculate the mutagenesis frequencies (% indels). Samples for which no uncut amplicon was detected are unlabeled. Control samples lacking TALEN expression are labeled (−). (B, E) Mutants recovered from *ADH1* and *NATA2b* TALEN plant lines. Representative T2 seedlings from the indicated parental T1 lines were screened by the PCR and restriction enzyme digestion assay. Uncut bands denote the presence of an inherited mutation. (C, F) Sequences of germinal mutations at *ADH1* and *NATA2* loci. Left and right TALEN target sequences are shaded in gray, with spacer sequences in lowercase. Deletion lengths are indicated to the right of the aligned sequences. The asterisk denotes a mutation that was identified in the somatic tissue of the T1 plant. Some mutations were identified in more than one mutant progeny, as indicated by the number in parentheses.

We analyzed 11 T1 plant lines transformed with the *ADH1* TALENs and identified four with high levels of somatic mutagenesis (Figure 3A). Mutagenesis frequencies in lines 1, 7, and 11 were 12%, 42%, and 33%, respectively. Line 6 had a lower frequency of 5%. Seed was collected from these lines, and individual T2 seedlings were screened for mutations by PCR amplification and restriction enzyme digestion. One germinal event was recovered from line 1, 3 events were recovered from line 7, and 1 event was recovered from line 11 (Figure 3B). The PCR amplicons were then sequenced to verify the presence of mutations in the TALEN spacer sequence. All T2 plants contained small deletions (2, 5, and 9 bp) within the spacer (Figure 3C). Thirty-four T2 seedlings from line 6 (low somatic mutagenesis) were also screened, but no germinal events were recovered. Line 7 had the highest number of germinal mutants (12%; 3 mutants out of 24 seedlings screened) and also had the highest frequency of somatic mutagenesis in the parental line (42%). The overall germinal transmission frequency of TALEN-induced mutations at the *ADH1* locus was ∼4% (Table 1). Seed from one of the *ADH1* mutants from line 7 was analyzed in the next generation (T3). Consistent with transmission of a Mendelian factor, we recovered heterozygous individuals (9 of 19 plants), homozygous mutants (3 of 19 plants), and homozygous wild-type plants (7 of 19 plants). Our data collectively indicate that TALEN-induced germinal mutations can be recovered at reasonably high frequencies using parental lines that efficiently express the TALENs.

**Table 1:** Germline transmission of TALEN-induced mutations in Arabidopsis

TALEN Target	T1 Line	Somatic Mutagenesis Levels (%)	Number of T2 Plants Screened	Number of Mutant Progeny Recovered	Transmission Frequency (%)*^a^*
*ADH1*	1	12	65	1	1.5
	6	5	14	0	0
	7	42	24	3	12.0
	11	33	30	1	3.3
*NATA2b*	10	73	93	4*^b^*	4.3
	15	31	96	0	0

TALEN, TAL effector nuclease.

a Transmission frequencies were calculated as the number of mutants recovered divided by the total number of progeny screened.

b The same mutation was found in all mutant plants recovered.

### Constitutive TALEN expression enhances somatic mutagenesis and creates germinally transmitted mutations

Previous work in our laboratory suggested that there are toxic effects associated with constitutive expression of some ZFNs in Arabidopsis; thus, an inducible expression system was adopted (Zhang *et al.* 2010). Because TALENs appear to be less toxic than ZFNs in other eukaryotic systems (Li *et al.* 2011; Mussolino and Cathomen 2011; Mussolino *et al.* 2011; Kim *et al.* 2013), we sought to constitutively express TALENs in Arabidopsis. We placed TALENs under the control of a strong cauliflower mosaic virus (CaMV) 35S promoter and assessed cleavage efficiency among individual T1 plants. We focused the analysis on the *NATA2* locus, which had not been previously targeted using engineered nucleases. Eighteen hygromycin-resistant T1 plants expressing the *NATA2b* TALEN pair were analyzed by the PCR and restriction enzyme digestion assay described. Evidence for NHEJ-induced mutations was detected in 14 of the 18 lines, with somatic mutagenesis frequencies reaching as high as 73% in line 10 (Figure 3D). Predicted insertion or deletion (indel) mutations were verified by sequencing PCR amplicons. Progeny from line 10 and line 15 (30% mutagenesis frequency) were screened for the presence of inherited mutations at the *NATA2b* target site (Figure 3E). We recovered 4 of 93 T2 plants (4%) from line 10 with mutations in the TALEN spacer sequence (Figure 3F, Table 1). No germline mutants were recovered from line 15. For one of the mutant plants derived from line 10, segregation of the mutation was followed in the next generation (T3). We recovered heterozygous individuals (18 of 42 plants), homozygous mutants (9 of 42 plants), and homozygous wild-type plants (15 of 42 plants), consistent with transmission of a Mendelian factor. Taken together, we demonstrate that constitutive promoters can be used to express some TALENs without any apparent toxic effects, enabling the recovery of heritable mutations.

### TALENs can delete tandemly duplicated genes in somatic cells

**Figure 4:**
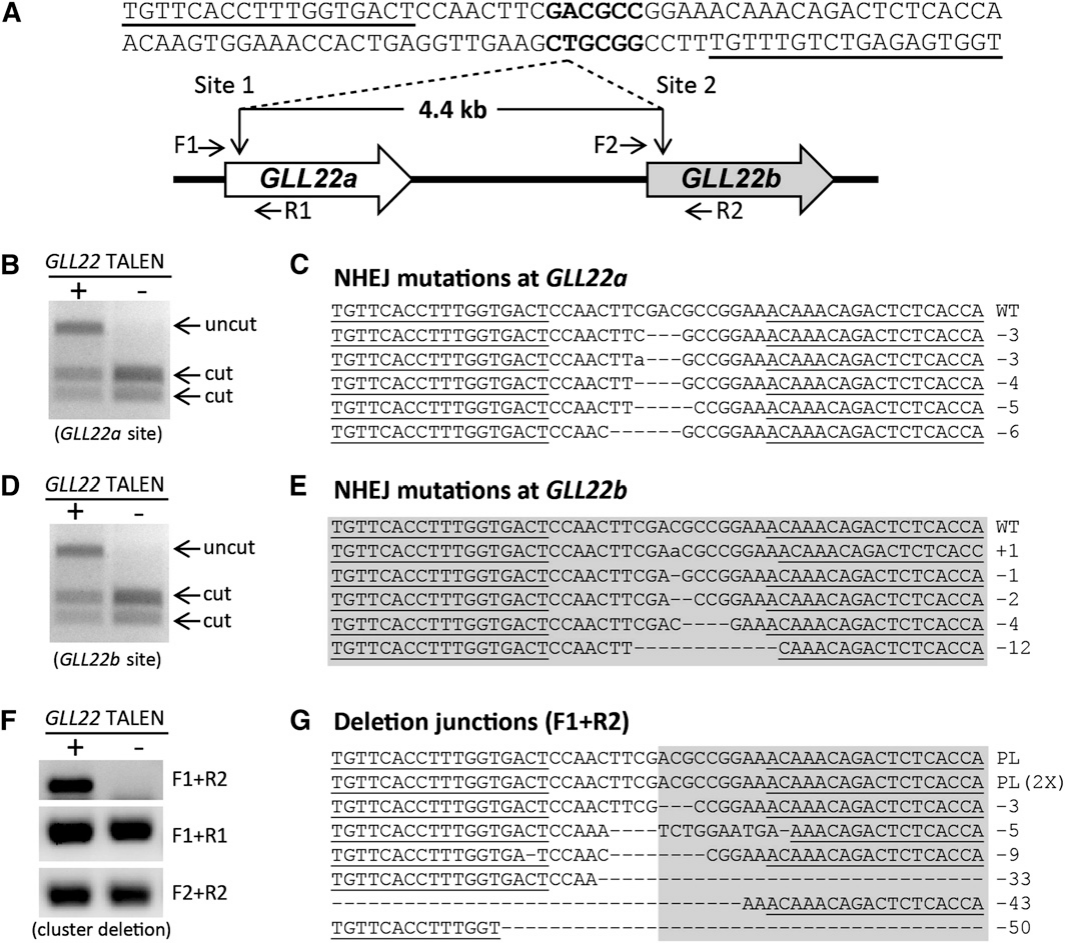
Targeted deletion of a *GLL22* gene cluster by TAL effector nucleases (TALENs) in somatic cells. (A) Schematic representation of the deletion of a gene cluster generated by the *GLL22* TALENs. TALEN binding sites in both gene copies are underlined, and the BsaHI restriction enzyme site in the spacer is in bold. (B) Nonhomologous end-joining (NHEJ)-induced mutagenesis activity of the *GLL22* TALEN pair at the *GLL22a* site. TALEN activity was assessed by the enrichment PCR assay. Either untreated (−) or TALEN-treated (+) genomic DNA was predigested with BsaHI, and the TALEN target site was PCR-amplified using primers unique to the region in *GLL22a*. Amplicons were digested again with BsaHI and separated on an agarose gel. (C) The uncut band in (B), representing mutated sequences, was cloned, and randomly selected clones were sequenced to reveal the mutations shown. (D) NHEJ-induced mutagenesis activity of the *GLL22* TALENs at the *GLL22b* site. The activity of *GLL22* TALEN at the *GLL22b* site was also assessed by enrichment PCR. (E) The resistant band generated by TALENs at the *GLL22b* site was cloned and randomly selected clones were sequenced as in (C). (F) Gene cluster deletion was confirmed by PCR with specific flanking primers (F1 and R2; A). PCR reactions amplifying both TALEN target sites were used as controls. (G) The deletion-specific PCR product was cloned and sequences were compared to the sequence of a perfect ligation (PL). One-week-old T1 transgenic (+) and Columbia WT (−) seedlings germinated on MS media with estradiol were used for this experiment.

To expand the utility and versatility of TALENs as tools for Arabidopsis genome modification, we assessed the ability of TALENs to target a duplicated gene array. Duplicated gene arrays are relatively common in many plant species, with nearly 17% of all genes in Arabidopsis arranged as tandem duplicates (The Arabidopsis Initiative 2000). We designed a TALEN pair to target the same sequence within two genes of a duplicated gene cluster whose DNA sequence is highly conserved. TALEN pair *GLL22* was designed to target and cleave two identical sequences within the tandem gene array *GLL22*, such that simultaneous cleavage of both cut sites might result in a deletion of 4.4 kb of intervening sequence (Figure 4A). TALEN expression was induced in T1 plants and cleavage efficiency at each target site was measured using enrichment PCR of pooled DNA samples.

The *GLL22* TALENs cleaved the predicted target sequence in each gene as evidenced by the fraction of uncut product in the TALEN-induced sample (Figure 4, B and D). Subsequent cloning and sequencing of the uncut fraction revealed expected indel mutations within the spacer of the TALEN target site in each gene (Figure 4, C and E). To determine if the TALEN pair could induce a large deletion, primers flanking the upstream TALEN target site *GLL22a* and the downstream target site *GLL22b* were used. Presence of an amplicon in the TALEN-treated (+) *vs.* untreated (−) sample indicated the expected deletion of 4.4 kb only in the treated sample. Sequence analysis of the deletion product showed expected deletion junctions between the two TALEN cut sites (Figure 4, F and G). Both perfect ligations and a range of deletions (3–50 bp) were recovered. These results demonstrate the feasibility of using TALENs to target repetitive gene clusters, as well as their potential to delete large spans of DNA between two target sites.

## Discussion

Here, we demonstrate targeted mutagenesis of endogenous Arabidopsis genes using TALENs. Key to recovery of heritable mutations was identifying plants with high frequencies of somatic mutagenesis. Plants that had somatic mutations in 5–73% of the *ADH1* and *NATA2* genes surveyed by PCR gave rise to mutant progeny at frequencies ranging from 4 to 12%.

Both optimal nuclease activity and high levels of TALEN expression were important in achieving efficient somatic mutagenesis. We found that the truncated N152/C63 TALEN architecture had higher activity in somatic cells than our original TALEN architecture, which has longer N-terminal and C-terminal extensions flanking the DNA binding domain (the so-called *Bam*HI architecture). When TAL effector DNA binding domains targeting the *ADH1* and *TT4* loci were tested in both architectures, *Bam*HI TALENs showed little *in vivo* activity (Figure S1B). The high activity of N152/C63 TALENs is consistent with our observations in tobacco, for which we compared a variety of TALEN N-terminal and C-terminal truncations to identify optimal architectures for activity in plants (Zhang *et al.* 2013).

Different lines of Arabidopsis with integrated TALEN constructs varied considerably in somatic mutagenesis frequencies, likely because of variable expression of the TALEN transgenes. This could be attributable to differences in copy number or influences from the genomic site of T-DNA integration (*i.e.*, position effects). Although all eight TALEN pairs tested in this study created mutations at their targets, the efficiencies ranged from 2 to 73% (Table S2). Transmission of TALEN-induced mutations to the next generation was observed only in progeny of T1 plants with the highest frequencies of somatic mutations, a correlation observed recently in zebrafish (Chen *et al.* 2013). Therefore, screening plants for high levels of somatic mutagenesis is recommended to recover heritable mutations.

Nearly all TALENs examined in this study showed comparable or even higher frequencies of somatic mutagenesis than ZFNs previously reported to generate heritable mutations in Arabidopsis (Lloyd *et al.* 2005; Osakabe *et al.* 2010; Zhang *et al.* 2010). Osakabe *et al.* (2010) reported ZFNs with relatively low somatic mutagenesis frequencies (0.26–3%), and yet heritable mutations were recovered in two of nine lines analyzed. We were not able to recover germinal mutants with comparably active TALENs. The *ADH1* and *TT4* TALENs induced mutations in somatic cell populations at frequencies nearly identical to two ZFNs targeting the same loci (5–15% for TALENs and 7–16% for ZFNs) (F. Zhang *et al.* 2010). However, transmission of TALEN-induced mutations to the next generation was considerably lower at the *ADH1* locus (69% for ZFNs *vs.* 12% for TALENs). For *TT4,* ZFNs induced mutations in 33% of progeny, whereas no mutations were recovered using the *TT4* TALENs. These results were unexpected considering that both the gene targets, method of nuclease expression, and levels of somatic mutagenesis were nearly identical between the two studies.

Is there an inherent difference between ZFNs and TALENs in their ability to generate heritable mutations in Arabidopsis? TAL effector proteins are derived from plant pathogens and, in some plants, TAL effectors trigger defense mechanisms (Boch and Bonas 2010). Thus, it is possible that a feature of the TALEN scaffold is recognized by the plant and this influences activity in the meristematic cells giving rise to the germline. Alternatively, the small size of ZFNs may allow for greater protein stability or expression in Arabidopsis germ cells. That said, we have also had difficulty in recovering germinal mutants for some ZFNs (Qi *et al.* 2013a). To date, the number of endogenous loci in Arabidopsis that have been successfully mutagenized with sequence-specific nucleases is low, and it remains to be determined if the observed differences in effectiveness of the two nuclease platforms will prove generalizable.

One difference in implementing TALEN-mediated mutagenesis in Arabidopsis *vs.* other models, including Drosophila, mice, rats, and human cells, is that the TALEN constructs are stably integrated into the genome. This so-called *in planta* approach presents a number of challenges in obtaining consistent results. As mentioned, one challenge derives from variable nuclease expression in the transgenic lines because of copy number variation and position effects. Additionally, T-DNA transfer can be incomplete, resulting in partial transgene integration, particularly at the right border (Rossi *et al.* 1996). We observed such incomplete insertions in several lines in which portions of the right TALEN were missing, and this correlates with the proportion of T1 plants with detectable NHEJ levels *vs.* the total number of hygromycin-resistant plants (Table S2).

A third challenge facing *in planta* mutagenesis strategies is that some nucleases are cytotoxic. Cytotoxicity with ZFNs led us to use the estrogen-inducible expression system to better control nuclease expression (F. Zhang *et al.* 2010; Qi *et al.* 2013b). TALENs have been reported to be less cytotoxic than ZFNs (Li *et al.* 2011; Mussolino and Cathomen 2011; Mussolino *et al.* 2011; Kim *et al.* 2013), and so we tested the constitutive CaMV 35S promoter, hoping to enhance mutagenesis frequencies. Primary transgenic plants constitutively expressing TALENs showed somatic mutagenesis efficiencies comparable with or higher than the estrogen-inducible TALENs (Table S2). Further, a higher proportion of plants with constitutively expressed TALENs had somatic efficiencies more than 10% relative to those regulated by estrogen. This was true across multiple targets (6 of 8 *vs.* 4 of 11 for *ADH1*; 9 of 18 *vs.* 2 of 9 for *NATA2b*). Although we did not observe any obvious toxic effects of the *NATA2b* TALEN pair that had somatic mutagenesis frequencies approximating 73%, for some TALEN pairs we obtained few or no primary transgenic plants, which may be attributed to cytotoxicity. This possible relationship between transformation efficiency and cytotoxicity, however, was not tested directly (Table S2). Clearly, there is still considerable room to optimize expression to maximize recovery of heritable mutations while simultaneously minimizing cytotoxic effects and off-target cleavage. A potential disadvantage of the 35S promoter is that it is weakly expressed in germ cells, and this could explain the lower-than-expected transmission frequency for the highly active *NATA2b* TALENs (Mascarenhas 1990). The use of the promoters such as EASE, which confines nuclease expression to the egg cell, may increase germinal recovery of targeted mutations (Even-Faitelson *et al.* 2011).

TALENs have been successfully used to modify the genomes of three additional plants, namely barley, rice, and Brachypodium (Li *et al.* 2012; Shan *et al.* 2013; Wendt *et al.* 2013). In all three species, tissue was transformed and plants regenerated that were resistant to the marker gene adjacent to the TALEN. In rice, approximately two-thirds of randomly selected T1 progeny carried monoallelic or biallelic mutations, suggesting that regeneration of plants directly from transformed cells may be a desirable alternative to *in planta* approaches, at least for plants amenable to cell culture and regeneration.

Our findings in Arabidopsis demonstrate that TALENs can be used to achieve high levels of mutagenesis in somatic cells, TALEN-mediated mutations can be recovered in the next generation from plants with high frequencies of somatic mutagenesis, and a single TALEN pair can be used to target a tandem gene array and create large deletions. It is expected that the work presented here will provide an easily adoptable framework for TALEN-mediated targeted mutagenesis in Arabidopsis, and future experiments will seek to optimize expression and better understand the relationship between somatic and germinal mutagenesis.

Note Added in Proof: See also Yiping Qi, Xiaohong Li, Yong Zhang, Colby G. Starker, Nicholas J. Baltes, Feng Zhang, Jeffry D. Sander, Deepak Reyon, J. Keith Joung, and Daniel F. Voytas, 2013 Targeted Deletion and Inversion of Tandemly Arrayed Genes in *Arabidopsis thaliana* Using Zinc Finger Nucleases G3: Genes, Genomes, Genetics 3: 1707–1715.

## Supplementary Material

Supporting Information

## References

[bib1] Alonso J. M. Stepanova A. N. Leisse T. J. Kim C. J. Chen H., P.*et al*, 2003 Genome-wide insertional mutagenesis of Arabidopsis thaliana. Science 301: 653–657 12893945 10.1126/science.1086391

[bib47] Bancroft I. Jones J. D. G. Dean C., 1993 Heterologous transposon tagging of the DRLl locus in Arabidopsis. The Plant Cell 5: 631–638 8392411 10.1105/tpc.5.6.631 PMC160301

[bib2] Boch J. Bonas U., 2010 Xanthomonas AvrBs3 family-type III effectors: discovery and function. Annu. Rev. Phytopathol. 48: 419–436 19400638 10.1146/annurev-phyto-080508-081936

[bib3] Cermak T. Doyle E. L. Christian M. Wang L. Zhang Y., 2011 Efficient design and assembly of custom TALEN and other TAL effector-based constructs for DNA targeting. Nucleic Acids Res. 39: e82 21493687 10.1093/nar/gkr218 PMC3130291

[bib4] Chen S. Oikonomou G. Chiu C. N. Niles B. J. Liu J., 2013 A large-scale in vivo analysis reveals that TALENs are significantly more mutagenic than ZFNs generated using context-dependent assembly. Nucleic Acids Res. 41: 1–10 23303782 10.1093/nar/gks1356 PMC3575824

[bib5] Christian M. Cermak T. Doyle E. L. Schmidt C. Zhang F., 2010 Targeting DNA double-strand breaks with TAL effector nucleases. Genetics 186: 757–761 20660643 10.1534/genetics.110.120717 PMC2942870

[bib6] Chuang C.-F. Meyerowitz E. M., 2000 Specific and heritable genetic interference by double-stranded RNA in Arabidopsis thaliana. Proc. Natl. Acad. Sci. USA 97: 4985–4990 10781109 10.1073/pnas.060034297 PMC18344

[bib7] Clough S. J. Bent A. F., 1998 Floral dip: a simplified method for Agrobacterium-mediated transformation of Arabidopsis thaliana. Plant J. 16: 735–743 10069079 10.1046/j.1365-313x.1998.00343.x

[bib8] Cong L. Ran F. A. Cox D. Lin S. Barretto R., 2013 Multiplex genome engineering using CRISPR/Cas systems. Science 339: 819–823 23287718 10.1126/science.1231143 PMC3795411

[bib9] Doyle E. L. Booher N. J. Standage D. S. Voytas D. F. Brendel V. P., 2012 TAL Effector-Nucleotide Targeter (TALE-NT) 2.0: tools for TAL effector design and target prediction. Nucleic Acids Res. 40: W117–22 22693217 10.1093/nar/gks608 PMC3394250

[bib10] Even-Faitelson L. Samach A. Melamed-Bessudo C. Avivi-Ragolsky N. Levy A., 2011 Localized egg-cell expression of effector proteins for targeted modification of the Arabidopsis genome. Plant J. 68: 929–937 21848915 10.1111/j.1365-313X.2011.04741.x

[bib11] Feldmann K. A., 1991 T-DNA insertion mutagenesis in Arabidopsis: mutational spectrum. Plant J. 1: 71–82

[bib12] Furner I. J. Pumfrey J. E., 1992 Cell fate in the shoot apical meristem of Arabidopsis thaliana. Development 115: 755–764

[bib13] Guschin D. Y. Waite A. J. Katibah G. E. Miller J. C. Holmes M. C., 2010 A rapid and general assay for monitoring endogenous gene modification. Methods Mol. Biol. 649: 247–256 20680839 10.1007/978-1-60761-753-2_15

[bib14] Halpin C. Cooke S. E. Barakate A. El Amrani A. Ryan M. D., 1999 Self-processing 2A-polyproteins - a system for co-ordinate expression of multiple proteins in transgenic plants. Plant J. 17: 453–459 10205902 10.1046/j.1365-313x.1999.00394.x

[bib15] Henikoff S. Till B. J. Comai L., 2004 TILLING: Traditional mutagenesis meets functional genomics. Plant Physiol. 135: 630–636 15155876 10.1104/pp.104.041061 PMC514099

[bib17] Kim H. J. H. Lee H. J. Kim H. J. H. Cho S. W. Kim J.-S., 2009 Targeted genome editing in human cells with zinc finger nucleases constructed via modular assembly. Genome Res. 19: 1279–1288 19470664 10.1101/gr.089417.108 PMC2704428

[bib18] Kim Y. Kweon J. Kim A. Chon J. K. Yoo J. Y., 2013 A library of TAL effector nucleases spanning the human genome. Nat. Biotechnol. 31: 251–258 23417094 10.1038/nbt.2517

[bib19] Li T. Huang S. Zhao X. Wright D. A. Carpenter S., 2011 Modularly assembled designer TAL effector nucleases for targeted gene knockout and gene replacement in eukaryotes. Nucleic Acids Res. 39: 6315–6325 21459844 10.1093/nar/gkr188 PMC3152341

[bib20] Li T. Liu B. Spalding M. H. Weeks D. P. Yang B., 2012 High-efficiency TALEN-based gene editing produces disease-resistant rice. Nat. Biotechnol. 30: 390–392 22565958 10.1038/nbt.2199

[bib21] Lloyd A. Plaisier C. L. Carroll D. Drews G. N., 2005 Targeted mutagenesis using zinc-finger nucleases in Arabidopsis. Proc. Natl. Acad. Sci. USA 102: 2232–2237 15677315 10.1073/pnas.0409339102 PMC548540

[bib22] Maeder M. L. Thibodeau-Beganny S. Osiak A. Wright D. A. Anthony R. M., 2008 Rapid “open-source” engineering of customized zinc-finger nucleases for highly efficient gene modification. Mol. Cell 31: 294–301 18657511 10.1016/j.molcel.2008.06.016 PMC2535758

[bib23] Mahfouz M. M. Li L. Shamimuzzaman M. Wibowo A. Fang X., 2010 De novo-engineered transcription activator-like effector (TALE) hybrid nuclease with novel DNA binding specificity creates double-strand breaks. Proc. Natl. Acad. Sci. USA 108: 2623–2628 21262818 10.1073/pnas.1019533108 PMC3038751

[bib24] Mali P. Yang L. Esvelt K. M. Aach J. Guell M., 2013 RNA-guided human genome engineering via Cas9. Science 339: 823–826 23287722 10.1126/science.1232033 PMC3712628

[bib25] Mascarenhas J. P., 1990 Gene activity during pollen development. Annu. Rev. Plant Physiol. Plant Mol. Biol. 41: 317–338

[bib26] McCallum C. M. Comai L. Greene E. A. Henikoff S., 2000 Targeted screening for induced mutations. Nat. Biotechnol. 18: 455–457 10748531 10.1038/74542

[bib27] Miller J. C. Tan S. Qiao G. Barlow K. A. Wang J., 2011 A TALE nuclease architecture for efficient genome editing. Nat. Biotechnol. 29: 143–148 21179091 10.1038/nbt.1755

[bib28] Mussolino C. Cathomen T., 2011 On target? Tracing zinc-finger-nuclease specificity. Nat. Methods 8: 725–726 21878917 10.1038/nmeth.1680

[bib29] Mussolino C. Morbitzer R. Lütge F. Dannemann N. Lahaye T., 2011 A novel TALE nuclease scaffold enables high genome editing activity in combination with low toxicity. Nucleic Acids Res. 39: 1–11 21813459 10.1093/nar/gkr597 PMC3241638

[bib30] Osakabe K. Osakabe Y. Toki S., 2010 Site-directed mutagenesis in Arabidopsis using custom-designed zinc finger nucleases. Proc. Natl. Acad. Sci. USA 107: 12034–12039 20508151 10.1073/pnas.1000234107 PMC2900650

[bib31] Qi Y. Li X. Zhang Y. Starker C. G. Baltes N. J. Zhang F. Sander J. D. Reyon D. Joung K. J. Voytas D. F., 2013 a Targeted deletion and inversion of tandemly arrayed genes in *Arabidopsis thaliana* somatic cells using zinc finger nucleases. G3 3: 1707–1715 23979943 10.1534/g3.113.006270 PMC3789795

[bib32] Qi Y. Zhang Y. Zhang F. Baller J. A. Cleland S. C., 2013 b Increasing frequencies of site-specific mutagenesis and gene targeting in Arabidopsis by manipulating DNA repair pathways. Genome Res. 23: 547–554 23282329 10.1101/gr.145557.112 PMC3589543

[bib33] Ramirez C. L. Foley J. E. Wright D. A. Müller-Lerch F. Rahman S. H., 2008 Unexpected failure rates for modular assembly of engineered zinc fingers. Nat. Methods 5: 374–375 18446154 10.1038/nmeth0508-374 PMC7880305

[bib34] Reyon D. Tsai S. Q. Khayter C. Foden J. A. Sander J. D., 2012 FLASH assembly of TALENs for high-throughput genome editing. Nat. Biotechnol. 30: 460–465 22484455 10.1038/nbt.2170 PMC3558947

[bib35] Rossi L. Hohn B. Tinland B., 1996 Integration of complete transferred DNA units is dependent on the activity of virulence E2 protein of Agrobacterium tumefaciens. Proc. Natl. Acad. Sci. USA 93: 126–130 8552588 10.1073/pnas.93.1.126 PMC40191

[bib36] Schneider C. A. Rasband W. S. Eliceiri K. W., 2012 NIH Image to ImageJ: 25 years of image analysis. Nat. Methods 9: 671–675 22930834 10.1038/nmeth.2089 PMC5554542

[bib37] Schwab R. Ossowski S. Riester M. Warthmann N. Weigel D., 2006 Highly specific gene silencing by artificial microRNAs in Arabidopsis. Plant Cell 18: 1121–1133 16531494 10.1105/tpc.105.039834 PMC1456875

[bib38] Shan Q. Wang Y. Chen K. Liang Z. Li J., 2013 Rapid and efficient gene modification in rice and Brachypodium using TALENs. Mol. Plant 6: 1–11 10.1093/mp/sss162 PMC3968307 23288864

[bib39] The Arabidopsis Initiative, 2000 Analysis of the genome sequence of the flowering plant *Arabidopsis thaliana*. Nature 408: 796–815 11130711 10.1038/35048692

[bib40] Voytas D. F., 2012 Plant genome engineering with sequence-specific nucleases. Annu. Rev. Plant Biol. 64: 327–350 23451779 10.1146/annurev-arplant-042811-105552

[bib41] Waterworth W. M. Drury G. E. Bray C. M. West C. E., 2011 Repairing breaks in the plant genome: the importance of keeping it together. New Phytol. 192: 805–822 21988671 10.1111/j.1469-8137.2011.03926.x

[bib42] Wendt T. Holm P. B. Starker C. G. Christian M. Voytas D. F., 2013 TAL effector nucleases induce mutations at a pre-selected location in the genome of primary barley transformants. Plant Mol. Biol. 10.1007/s11103–013–0078–4 10.1007/s11103-013-0078-4 PMC7880306 23689819

[bib43] Zhang F. Maeder M. L. Unger-Wallace E. Hoshaw J. P. Reyon D., 2010 High frequency targeted mutagenesis in Arabidopsis thaliana using zinc finger nucleases. Proc. Natl. Acad. Sci. USA 107: 12028–12033 20508152 10.1073/pnas.0914991107 PMC2900673

[bib44] Zhang F. Cong L. Lodato S. Kosuri S. Church G. M., 2011 Efficient construction of sequence-specific TAL effectors for modulating mammalian transcription. Nat. Biotechnol. 29: 149–153 21248753 10.1038/nbt.1775 PMC3084533

[bib45] Zhang Y. Zhang F. Li X. Baller J. A. Qi Y., 2013 Transcription activator-like effector nucleases enable efficient plant genome engineering. Plant Physiol. 161: 20–27 23124327 10.1104/pp.112.205179 PMC3532252

[bib46] Zuo J. Niu Q. W. Chua N. H., 2000 Technical advance: An estrogen receptor-based transactivator XVE mediates highly inducible gene expression in transgenic plants. Plant J. 24: 265–273 11069700 10.1046/j.1365-313x.2000.00868.x

