# Supplementary material for: Targeted Mutagenesis of Arabidopsis thaliana Using Engineered TAL Effector Nucleases
Source: G3 (Bethesda). 2013 Oct 1;3(10):1697–705. doi: 10.1534/g3.113.007104 (PMC3789794; doi:10.1534/g3.113.007104)
Supplement: Supporting Information [file supp_3_10_1697__index.html]

Targeted Mutagenesis of Arabidopsis thaliana Using Engineered TAL Effector Nucleases — Supporting Information 

# Targeted Mutagenesis of *Arabidopsis thaliana* Using Engineered TAL Effector Nucleases

## Supporting Information for Christian *et al.*

**Files in this Data Supplement:**

- Supporting Information - Figure S1 and Tables S1-S2 (PDF, 2 MB)
- Figure S1 - Activity of BamHII TALENs in yeast and at endogenous targets in *Arabidopsis* somatic cells (PDF, 1 MB)
- Table S1 - Information on engineered TALENs (PDF, 91 KB)
- Table S2 - Summary of TALEN activity in *Arabidopsis* (PDF, 99 KB)
