# Supplementary material for: Targeted Mutagenesis of Arabidopsis thaliana Using Engineered TAL Effector Nucleases
Source: G3 (Bethesda). 2013 Oct 1;3(10):1697–705. doi: 10.1534/g3.113.007104 (PMC3789794; doi:10.1534/g3.113.007104)
Supplement: Supporting Information [file supp_g3.113.007104_007104SI.pdf]

G3: Genes | Genomes | Genetics

Targeted Mutagenesis of *Arabidopsis thaliana* using Engineered TAL Effector Nucleases (TALENs)

Michelle Christian[*,1], Yiping Qi[*,1], Yong Zhang[§] and Daniel F. Voytas[*,2]

[*]Department of Genetics, Cell Biology & Development and Center for Genome Engineering, University of Minnesota, Minneapolis, MN 55455
[§]Department of Biotechnology, School of Life Sciences and Technology, University of Electronic Science and Technology of China, Chendu 610054, China

DOI: 10.1534/g3.113.007104

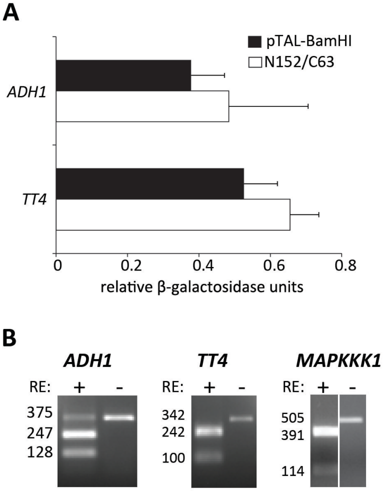

C *ADH1*-BamHI TALEN target

```
CCGGATGCTCCTCTTGACAAGGTCTGTATTGTCAGTTGTGGTTTGTCT WT
CCGGATGCTCCTCTTGACAAGG---GTATTGTCAGTTGTGGTTTGTCT -3
CCGGATGCTCCTCTTGACAAGG----TATTGTCAGTTGTGGTTTGTCT -4
CCGGATGCTCCTCTTGACAAG-----TATTGTCAGTTGTGGTTTGTCT -5
CCGGATGCTCCTCTTGACAAGGc-----TTGTCAGTTGTGGTTTGTCT -5/+1
CCGGATGCTCCTCTTGACAAGG-----ATTGTCAGTTGTGGTTTGTCT -5
CCGGATGCTCCTCTTGACAAG------ATTGTCAGTTGTGGTTTGTCT -6
CCGGATGCTCCTCTTGACAA-------ATTGTCAGTTGTGGTTTGTCT -7
CCGGATGCTCCTCTTa-----------ATTGTCAGTTGTGGTTTGTCT -11/+1
CCGGATGCTCCTCTT--------------TGTCAGTTGTGGTTTGTCT -14
CCGGATGCTCCTCTTGACAAGG-------------------TTTGTCT -19
CCGGATG-----------------------------------GTTTGTCT -33
```

Figure S1. Activity of BamHII TALENs in yeast and at endogenous targets in *Arabidopsis* somatic cells*.* (A) Singlen strand annealing (SSA) assays in yeast show high TALEN activity irrespective of architecture. Briefly, *ADH1* and *TT4* TALEN pairs with either the pTALn BamHI backbone architecture (black bars) or truncated N152/C63 architecture (white bars) were transformed to yeast of one mating type. Cognate target plasmids harboring a disrupted *lacZ* gene were transformed into yeast of the opposite mating type. Upon mating, active TALENs cleaved their cognate targets resulting in restoration of *lacZ,* which was measured by bn galactosidase enzyme assays (Christian, Cermak, Doyle, Schmidt, Zhang, Hummel, et al., 2010). Values are normalized to a TALEN positive control and expressed as the mean of duplicates with standard deviation. (B) Analysis of pTALn BamHI TALEN activity by enrichment PCR. Genomic DNA from 5n 10 pooled seedlings for each BamHIn TALEN was digested with a restriction enzyme whose site is present in the TALEN spacer sequence (see Figure 2A). The restriction digestions were PCRn amplified and digested a second time. Presence of a resistant band in the digested (+) TALEN sample versus undigested (n) sample indicates targeted mutations. Fully digested products in the (+) sample indicate absence of TALENn induced mutations. (C) Sequences of mutations induced by the *ADH1* pTALn BamHI TALENs. Clones derived from the undigested product in panel B were sequenced. Left and right TALEN target sequences are underlined in the WT sequence; gray shading denotes the TALEN target sequence in the clones. Deletions in the sequences alignments are represented by dashes and inserted bases are lowercased. The lengths of insertions (+) or deletions (n) are indicated to the right of the sequences.

Table S1. Information on engineered TALENs.

TALEN ID	Gene name	Repeat number	Spacer length	RVDs	Target sequence
ADH1 left	*ADH1*	15	18	HD HD NN NN NI NG NN HD NG HD HD NG HD NG NG	CCGGATGCTCCTCTT GACAAGGTCTGTATTGT C AGTTGTGGTTTGTCT
ADH1 right		15		NI NN NI HD NI NI NI HD HD NI HD NI NI HD NG	
TT4 left	*TT4*	15	15	NN NG HD NN NG HD NG NG HD NG NN HD NI HD NG	GTCGTCTTCTGCACT ACCTCCGGCGTCGAC ATGCCTGGTGCTGACT
TT4 right		16		NI NN NG HD NI NN HD NI HD HD NI NN NN HD NI NG	
MAPKKK1 left	*MAPKKK1*	17	14	NI NI NG NG NN NN NG HD NN HD NN NN HD NN HD NN NG	AATTGGTCGCGGCGCGT TTGGTACGGTGTAC ATGGGTATGAATCTTG
MAPKKK1 right		16		HD NI NI NN NI NG NG HD NI NG NI HD HD HD NI NG	
DSK2Ba left	*DSK2B* (exon 1)	17	22	NI NN NG NN NG NN NI NI NN NI HD NN NI NN NG HD NG	AGTGTGAAGACGAGTCT CGATTCAACGGTGGAGT CTTTCAAAGAGTTGGTG GCTC
DSK2Ba right		16		NN NI NN HD HD NI HD HD NI NI HD NG HD NG NG NG	
DSK2Bb left	*DSK2B* (exon 2)	18	17	NN NI HD HD HD NI NI NN HD NI NG HD HD NG NG HD NN NG	GACCCAAGCATCCTTCG TCAAACTCTAGAAGCGG CAAGAAACCCAGAGCTT
DSK2Bb right		16		NI NI NN HD NG HD NG NN NN NN NG NG NG HD NG NG	
NATA2a left	*NATA1*	15	18	HD NN NN HD HD NI HD HD HD NI NI NG NN NG NG	CGGCCACCCAATGTT CTCCCGGATCCGTCTGG CAACACCGTCCGATGTC CC
NATA2a right	(exon 1)	18		NN NN NN NI HD NI NG HD NN NN NI HD NN NN NG NN NG NG	
NATA2b left	*NATA1*	15	18	NI NN NN NI NN NN NI NI NI NN NN NN NG NG NG	AGGAGGAAAGGGTTT GGTAGCATGTTGTTG ACTGCTGTGGCCAAAC
NATA2b right	(exon 1)	16		NN NG NG NG NN NN HD HD NI HD NI NN HD NI NN NG	
GLL22 left	*GLL22 (At1g54000 At1g54010)*	18	18	NG NN NG NG HD NI HD HD NG NG NG NN NN NG NN NI HD NG	TGTTCACCTTTGGTGACT CCAACTTCGACGCCGGA AACAAACAGACTCTCAC CA
GLL22 right		18		NG NN NN NG NN NI NN NI NN NG HD NG NN NG NG NG NN NG	

Table S2. Summary of TALEN activity in *Arabidopsis.*

TALEN target	Expression method	Number of parental T1 plants with somatic indels (total transgenic)	Somatic NHEJ efficiency	Number of progeny screened	Number of mutant progeny recovered
ADH1	XVE	6 (13)	5 – 42%	133	5
ADH1	35S	6 (8)	10 – 60%	nt	_
TT4	XVE	1 (3)[a]	6 – 7%	134	0
MAPKKK1	XVE	1 (4)[a]	5%	108	0
DSK2Ba	XVE	5 (10)	3 – 9%	386	0
DSK2Bb	XVE	4 (6)	2.5 – 7%	248	0
NATA2a	XVE	10 (13)	2.5 – 28%	99	0
NATA2a	35S	2 (6)	nt	60	0
NATA2b	XVE	6 (9)	4 – 27%	172	0
NATA2b	35S	14 (18)	2 – 73%	189	4

nt = not tested.

[a] Consistently low recovery of transgenic plants over three separate transformation experiments.
