# Supplementary material for: Targeted Mutagenesis of Arabidopsis thaliana Using Engineered TAL Effector Nucleases
Source: G3 (Bethesda). 2013 Oct 1;3(10):1697–705. doi: 10.1534/g3.113.007104 (PMC3789794; doi:10.1534/g3.113.007104)
Supplement: Supporting Information [file supp_g3.113.007104_FigureS1.pdf]

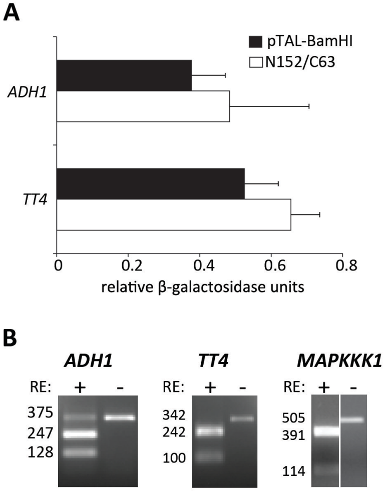

C *ADH1*-BamHI TALEN target

```
CCGGATGCTCCTCTTGACAAGGTCTGTATTGTCAGTTGTGGTTTGTCT WT
CCGGATGCTCCTCTTGACAAGG---GTATTGTCAGTTGTGGTTTGTCT -3
CCGGATGCTCCTCTTGACAAGG----TATTGTCAGTTGTGGTTTGTCT -4
CCGGATGCTCCTCTTGACAAG-----TATTGTCAGTTGTGGTTTGTCT -5
CCGGATGCTCCTCTTGACAAGGc-----TTGTCAGTTGTGGTTTGTCT -5/+1
CCGGATGCTCCTCTTGACAAGG-----ATTGTCAGTTGTGGTTTGTCT -5
CCGGATGCTCCTCTTGACAAG------ATTGTCAGTTGTGGTTTGTCT -6
CCGGATGCTCCTCTTGACAA-------ATTGTCAGTTGTGGTTTGTCT -7
CCGGATGCTCCTCTTa-----------ATTGTCAGTTGTGGTTTGTCT -11/+1
CCGGATGCTCCTCTT--------------TGTCAGTTGTGGTTTGTCT -14
CCGGATGCTCCTCTTGACAAGG-------------------TTTGTCT -19
CCGGATG---------------------------------GTTTGTCT -33
```

Figure S1. Activity of BamHII TALENs in yeast and at endogenous targets in *Arabidopsis* somatic cells*.* (A) Singlen strand annealing (SSA) assays in yeast show high TALEN activity irrespective of architecture. Briefly, *ADH1* and *TT4* TALEN pairs with either the pTALn BamHI backbone architecture (black bars) or truncated N152/C63 architecture (white bars) were transformed to yeast of one mating type. Cognate target plasmids harboring a disrupted *lacZ* gene were transformed into yeast of the opposite mating type. Upon mating, active TALENs cleaved their cognate targets resulting in restoration of *lacZ,* which was measured by bn galactosidase enzyme assays (Christian, Cermak, Doyle, Schmidt, Zhang, Hummel, et al., 2010). Values are normalized to a TALEN positive control and expressed as the mean of duplicates with standard deviation. (B) Analysis of pTALn BamHI TALEN activity by enrichment PCR. Genomic DNA from 5n 10 pooled seedlings for each BamHIn TALEN was digested with a restriction enzyme whose site is present in the TALEN spacer sequence (see Figure 2A). The restriction digestions were PCRn amplified and digested a second time. Presence of a resistant band in the digested (+) TALEN sample versus undigested (n) sample indicates targeted mutations. Fully digested products in the (+) sample indicate absence of TALENn induced mutations. (C) Sequences of mutations induced by the *ADH1* pTALn BamHI TALENs. Clones derived from the undigested product in panel B were sequenced. Left and right TALEN target sequences are underlined in the WT sequence; gray shading denotes the TALEN target sequence in the clones. Deletions in the sequences alignments are represented by dashes and inserted bases are lowercased. The lengths of insertions (+) or deletions (n) are indicated to the right of the sequences.
