# Supplementary material for: Targeted Mutagenesis of Arabidopsis thaliana Using Engineered TAL Effector Nucleases
Source: G3 (Bethesda). 2013 Oct 1;3(10):1697–705. doi: 10.1534/g3.113.007104 (PMC3789794; doi:10.1534/g3.113.007104)
Supplement: Supporting Information [file supp_g3.113.007104_TableS1.pdf]

Table S1. Information on engineered TALENs.

TALEN ID	Gene name	Repeat number	Spacer length	RVDs	Target sequence
ADH1 left	*ADH1*	15	18	HD HD NN NN NI NG NN HD NG HD HD NG HD NG NG	CCGGATGCTCCTCTT GACAAGGTCTGTATTGT C AGTTGTGGTTTGTCT
ADH1 right		15		NI NN NI HD NI NI NI HD HD NI HD NI NI HD NG	
TT4 left	*TT4*	15	15	NN NG HD NN NG HD NG NG HD NG NN HD NI HD NG	GTCGTCTTCTGCACT ACCTCCGGCGTCGAC ATGCCTGGTGCTGACT
TT4 right		16		NI NN NG HD NI NN HD NI HD HD NI NN NN HD NI NG	
MAPKKK1 left	*MAPKKK1*	17	14	NI NI NG NG NN NN NG HD NN HD NN NN HD NN HD NN NG	AATTGGTCGCGGCGCGT TTGGTACGGTGTAC ATGGGTATGAATCTTG
MAPKKK1 right		16		HD NI NI NN NI NG NG HD NI NG NI HD HD HD NI NG	
DSK2Ba left	*DSK2B* (exon 1)	17	22	NI NN NG NN NG NN NI NI NN NI HD NN NI NN NG HD NG	AGTGTGAAGACGAGTCT CGATTCAACGGTGGAGT CTTTCAAAGAGTTGGTG GCTC
DSK2Ba right		16		NN NI NN HD HD NI HD HD NI NI HD NG HD NG NG NG	
DSK2Bb left	*DSK2B* (exon 2)	18	17	NN NI HD HD HD NI NI NN HD NI NG HD HD NG NG HD NN NG	GACCCAAGCATCCTTCG TCAAACTCTAGAAGCGG CAAGAAACCCAGAGCTT
DSK2Bb right		16		NI NI NN HD NG HD NG NN NN NN NG NG NG HD NG NG	
NATA2a left	*NATA1*	15	18	HD NN NN HD HD NI HD HD HD NI NI NG NN NG NG	CGGCCACCCAATGTT CTCCCGGATCCGTCTGG CAACACCGTCCGATGTC CC
NATA2a right	(exon 1)	18		NN NN NN NI HD NI NG HD NN NN NI HD NN NN NG NN NG NG	
NATA2b left	*NATA1*	15	18	NI NN NN NI NN NN NI NI NI NN NN NN NG NG NG	AGGAGGAAAGGGTTT GGTAGCATGTTGTTG ACTGCTGTGGCCAAAC
NATA2b right	(exon 1)	16		NN NG NG NG NN NN HD HD NI HD NI NN HD NI NN NG	
GLL22 left	*GLL22 (At1g54000 At1g54010)*	18	18	NG NN NG NG HD NI HD HD NG NG NG NN NN NG NN NI HD NG	TGTTCACCTTTGGTGACT CCAACTTCGACGCCGGA AACAAACAGACTCTCAC CA
GLL22 right		18		NG NN NN NG NN NI NN NI NN NG HD NG NN NG NG NG NN NG	
