# Supplementary material for: Targeted Mutagenesis of Arabidopsis thaliana Using Engineered TAL Effector Nucleases
Source: G3 (Bethesda). 2013 Oct 1;3(10):1697–705. doi: 10.1534/g3.113.007104 (PMC3789794; doi:10.1534/g3.113.007104)
Supplement: Supporting Information [file supp_g3.113.007104_TableS2.pdf]

Table S2. Summary of TALEN activity in *Arabidopsis*.

TALEN target	Expression method	Number of parental T1 plants with somatic indels (total transgenic)	Somatic NHEJ efficiency	Number of progeny screened	Number of mutant progeny recovered
ADH1	XVE	6 (13)	5 – 42%	133	5
ADH1	35S	6 (8)	10 – 60%	nt	_
TT4	XVE	1 (3)[a]	6 – 7%	134	0
MAPKKK1	XVE	1 (4)[a]	5%	108	0
DSK2Ba	XVE	5 (10)	3 – 9%	386	0
DSK2Bb	XVE	4 (6)	2.5 – 7%	248	0
NATA2a	XVE	10 (13)	2.5 – 28%	99	0
NATA2a	35S	2 (6)	nt	60	0
NATA2b	XVE	6 (9)	4 – 27%	172	0
NATA2b	35S	14 (18)	2 – 73%	189	4

nt = not tested.
[a] Consistently low recovery of transgenic plants over three separate transformation experiments.
